# Spontaneous Coronary Sinus Thrombosis Detected by Point-of-care Transthoracic Echo: A Case Report

**DOI:** 10.5811/cpcem.1590

**Published:** 2023-08-04

**Authors:** Lily Leitner Berrin, Kaitlen Howell, Amanda Foote, Jordan Mullings, Akash Desai, Martha Montgomery, Sophie Barbant, Arun Nagdev

**Affiliations:** * Highland Hospital, Alameda Health System, Department of Emergency Medicine, Oakland, California; † University of California San Francisco, Department of Emergency Medicine, San Francisco, California; ‡ Highland Hospital, Alameda Health System, Department of Cardiology, Oakland, California

**Keywords:** Case report, POCUS, TTE, coronary sinus thrombosis, ECG

## Abstract

**Introduction:**

Coronary sinus thrombosis (CST) is a rare condition, primarily occurring after instrumentation of the heart, with no prior reported cases diagnosed via point-of-care ultrasound or of spontaneous occurrence without predisposing medical or surgical history. Patients typically present with critical illness, and CST has a reported mortality of 80%.

**Case Report:**

We present a case of a healthy 38-year-old male with chest pain one hour after cocaine use, with an electrocardiogram pattern consistent with Wellens syndrome, whose point-of-care cardiac ultrasound revealed CST.

**Conclusion:**

This uncommon ultrasonographic finding has never been reported in the emergency medicine literature to our knowledge. It can be recognized by the clinician sonographer during standard point-of-care transthoracic echocardiogram.

## INTRODUCTION

Coronary sinus thrombosis (CST) refers to clot formation within the blood vessel that drains most of the coronary arteries’ deoxygenated blood. This vessel travels posterior to the junction of the left atrium and left ventricle before emptying directly into the right atrium. Coronary sinus thrombosis occurs as a rare complication of intracardiac instrumentation and very rarely due to infective endocarditis, congenital structural abnormalities, myocardial infarction, or prothrombotic states, with no previously reported cases occurring without predisposing medical or surgical history or in the setting of substance use.^1^ Patients typically present with critical illness and have a poor prognosis, with a reported mortality of 80%.^2^ Here we describe a case of CST detected by point-of-care ultrasound (POCUS) in a previously healthy, 38-year-old male presenting with chest pain within hours of cocaine and heroin use, associated with a Wellens pattern on electrocardiogram (ECG).^3^

## CASE REPORT

A 38-year-old male with a past medical history only of substance use disorder presented to the emergency department (ED) 20 minutes following intranasal stimulant use. The patient complained of severe chest pain that was sudden in onset, non-exertional, and non-radiating. He also reported mild shortness of breath and anxiety. Prehospital responders noted initial relief of his chest pain and shortness of breath following administration of 325 milligrams (mg) of aspirin and 0.4 mg of sublingual nitroglycerin, but the pain returned upon arrival to the ED. The patient’s initial vital signs at time of presentation included a blood pressure of 156/108 millimeters of mercury (mmHg), heart rate of 79 beats per minute, respiratory rate of 18 breaths per minute, and oxygen saturation of 100% on room air. Upon arrival, the patient reported chest pain and fatigue and denied any other symptoms. He also denied any family history of cardiac disease.

**Image 1:**
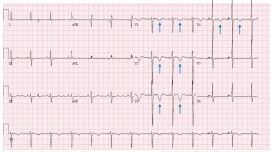
Initial echocardiogram with deep and symmetric T-wave inversions in leads V1–V4 (blue arrows), with no Q-waves, normal R-wave progression, and no ST-segment elevations, concerning for Wellens syndrome.

On physical examination, the patient appeared drowsy and had symmetrically small but not pinpoint, equally round and reactive pupils. Cardiopulmonary exam and complete physical examination were unremarkable. The emergency clinician obtained an ECG, chest radiograph, troponin level, complete blood count, comprehensive metabolic panel, and a bedside transthoracic echocardiogram (TTE) to guide medical management. The initial electrocardiogram ECG (Image 1) revealed deep and symmetric T-wave inversions in leads V1–V4, with no Q-waves, normal R-wave progression, and no ST-segment elevations, concerning for Wellens syndrome.

**Image 2:**
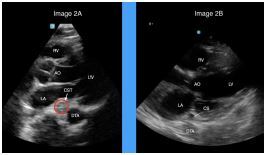
(A) Initial parasternal long-axis view of patient with coronary sinus thrombosis (CST); (B) normal coronary sinus (CS) with anatomic labels. *LA*, left atrium, *LV*, left ventricle, *AO*, aortic outflow; *RV*, right ventricle; *DTA*, descending thoracic aorta.

A cardiology consultation was initiated, and point-of-care TTE was then performed by emergency clinicians at point of care. On parasternal long view, a circular, hyperechoic focus was noted posterior to the left atrium, at the location of the coronary sinus (Image 2A). This can be compared to a parasternal long TTE view with a normal coronary sinus (Image 2B). There were no signs of systolic or diastolic dysfunction, no noted pericardial effusion, and the size of the atria and ventricles were within normal limits. At the time the echo was completed, the patient was resting comfortably in the gurney without chest pain.

Results from the patient’s blood tests came back unremarkable, including a normal troponin. Urine toxicology screen was positive for cocaine and opioids and negative for amphetamines. A chest radiograph was within normal limits. A comprehensive echocardiogram was subsequently performed and confirmed the diagnosis of CST. Because the patient was pain free, hemodynamically stable, and with negative serial troponins, cardiology recommended inpatient monitoring and anticoagulation, and did not recommend clot retrieval.

## DISCUSSION

The occurrence of CST is exceedingly rare. Most instances occur as a complication of right heart instrumentation, infective endocarditis, hypercoagulable states, or congenital malformation.^4^ This is the first reported case of CST detected by POCUS in an ED setting.

**CPC-EM Capsule**

What do we already know about this clinical entity?

*Coronary sinus thrombosis (CST) is a rare condition primarily occurring after instrumentation of the heart, with an 80% mortality rate.*

What makes this presentation of disease reportable?

*This is the first report of CST detected via point-of-care-ultrasound (POCUS) in a patient with no predisposing history but with recent cocaine use.*

What is the major learning point?

*Point-of-care-ultrasound is a valuable bedside tool that can detect rare conditions and track a dynamic pathologic process such as CST.*

How might this improve emergency medicine practice?

*Subtle abnormalities on POCUS can suggest possible uncommon pathological conditions. Combined with consultative echocardiography it can be useful in difficult cases.*

While cocaine is classically associated with coronary artery vasospasm and arrhythmia, its thrombogenic effects can be just as lethal.^5^,^6^ Cocaine induces a prothrombotic state by promoting platelet activity and aggregation, increasing clotting factor concentrations, and inhibiting thrombolysis.^7^ Previous reports discuss various cocaine-induced thromboses including deep vein thrombosis and pulmonary embolism, myocardial infarction from intracoronary thrombus despite normal coronary arteries, and floating aortic arch thrombus.^8^–^10^ Our case suggests CST may similarly be triggered by cocaine use.

Coronary sinus thrombosis may be acute or chronic and typically presents as a severe condition, with signs and symptoms including chest pain, shortness of breath, and hypotension.^4^ Secondary complications of CST may include pericardial effusion, cardiogenic shock, or even sudden cardiac death.^4^,^11^ More insidious presentations, including entirely asymptomatic cases, have been reported in partial or incomplete thrombus formation. Most often, CST is reported to be a fatal condition, with most cases identified via autopsy.^4^ Remarkably, the patient we present was well-appearing and hemodynamically stable, with resolved chest pain at the time POCUS echocardiography demonstrated CST. It is possible that CST is underdiagnosed in stable patients presenting with chest pain who are discharged from the ED and do not receive comprehensive echocardiography during their evaluation, highlighting the importance of POCUS and awareness of this sonographic diagnosis.

To date, POCUS has not been a common diagnostic tool for CST; in fact, we were unable to identify any prior published reports of point-of-care imaging used to diagnose CST. Previously, CST has been diagnosed using comprehensive echocardiography from either a transthoracic or transesophageal approach, with transesophageal views being more sensitive. Unlike comprehensive echocardiography performed by ultrasound technologists, POCUS allows for easy serial sonographic evaluation. Our consulting cardiologist reviewed and found dynamic changes between the initial POCUS study, a comprehensive echocardiogram obtained two hours later, and a repeat POCUS performed approximately four hours after the initial one. While the first images showed complete occlusion of the coronary sinus, the comprehensive study showed a slightly smaller thrombus with some flow present around the thrombus on color Doppler. The images obtained at four hours showed an even smaller thrombus within the coronary sinus. These progressive findings over hours may have increased the likelihood of this patient’s CST being related to his reported substance use that same day.

Dynamic ECG changes such as ST-segment elevations and left axis deviation can be found in acute CST, most likely due to increased myocardial perfusion pressure and decreased coronary artery blood flow leading to acute ischemia of the myocardium.^4^,^12^ To our knowledge, this is the first reported case of Wellens pattern associated with acute CST. However, it should be noted that the Wellens pattern on ECG may have been due to the patient’s recent cocaine use, independent of the CST.

There are no current guidelines for the management of CST, and treatment has varied by case given the rarity of the condition.^4^,^13^ Treatment options include interventional management with thrombectomy and medical management with anticoagulation. Thrombectomy followed by heparin as a bridge to warfarin therapy has been previously documented when a patient is critically ill or unstable.^14^ Other case reports include the use of low-molecular-weight heparin as a bridge to warfarin therapy or the use of novel anticoagulants, without clot retrieval, in clinically stable patients.^2^

In a case report detailing CST as a complication of ventricular free-wall rupture following a myocardial infarction, the risk of hemorrhage into the pericardium was deemed too great, and the patient was managed without anticoagulation, ultimately doing well with conservative management.^15^ The morbidity and mortality benefits of these varied approaches have not yet been established in the literature, and further clinical studies are needed. Ultimately, our patient was discharged on rivaroxaban after a short and uncomplicated hospital stay, with recommended repeat TTE in two months.

## CONCLUSION

Coronary sinus thrombosis is a rare condition that has never before been reported as detected by POCUS. Classically, point-of-care TTE is used for the assessment of gross left ventricular systolic function, detection of a pericardial effusion, and evaluation for right heart strain. As clinicians become more comfortable with performing these evaluations, other subtle and less common findings will be detected. Point-of-care ultrasound is a valuable bedside assessment tool for patients with chest pain and ECG changes and can be used to track a dynamic pathologic process such as CST. As in our case, the ECG pattern, along with the detection of a hyperechoic lesion in the location of the coronary sinus, triggered specialist consultation, comprehensive TTE, and therapeutic intervention.
